# Age-related variation in the oral microbiome of urban Cooper’s hawks (*Accipiter cooperii*)

**DOI:** 10.1186/s12866-019-1413-y

**Published:** 2019-02-21

**Authors:** Michael J. Taylor, R. William Mannan, Jana M. U’Ren, Nicholas P. Garber, Rachel E. Gallery, A. Elizabeth Arnold

**Affiliations:** 1 School of Natural Resources and the Environment, The University of Arizona, Tucson, USA; 2 Department of Biosystems Engineering, The University of Arizona, Tucson, USA; 3 School of Plant Sciences, The University of Arizona, Tucson, USA; 4 Department of Ecology and Evolutionary Biology, The University of Arizona, Tucson, USA

**Keywords:** 16S rRNA, Accipitridae, Bacteria, *Porphyromonas*, pH, Raptors, *Trichomonas*

## Abstract

**Background:**

Bird species worldwide are affected by trichomoniasis caused by the protist *Trichomonas gallinae*. In avivorous raptors such as Cooper’s hawks (*Accipiter cooperii*), nestlings are more susceptible than fledglings and adults. Previous research suggested a link between oral pH and susceptibility: the oral pH of fledgling and adult hawks is more than seven times more acidic than that of nestlings. We speculated that this age-specific difference in pH would correspond to age-specific differences in the oral microbiota of Cooper’s hawks. We examined the oral microbiomes of 31 healthy, wild Cooper’s hawks in Tucson, Arizona (USA). Individuals represented three age classes (nestlings, fledglings, and adults). We designed our study with multiple controls, replicated sampling, mock communities, and stringent quality-controls to address challenges that can limit the inferential quality of microbiome data sets.

**Results:**

Richness of bacterial communities in oral cavities of Cooper’s hawks differed as a function of age but not as a function of sex, sampling date, or sampling location. Bacterial communities in oral cavities of nestlings differed from those of fledglings and adults, whereas communities in fledglings and adults did not differ from each other. Communities were similar in males and females and did not differ over the sampling season. Prevalence of acid-producing bacteria in fledgling and adults vs. nestlings is consistent with previous reports of age-specific variation in oral pH, but further research is needed to establish a causal link to pH levels or susceptibility to disease. Analyses of mock communities demonstrated high repeatability and showed that operon number and read abundance were highly correlated.

**Conclusions:**

The oral microbiota of wild Cooper’s hawks differs between nestlings and older birds. Variation in the oral microbiome is consistent with differences in oral pH between nestlings and older individuals. Overall our study provides a first perspective on bacterial communities associated with oral cavities of a wild raptor.

**Electronic supplementary material:**

The online version of this article (10.1186/s12866-019-1413-y) contains supplementary material, which is available to authorized users.

## Background

Bird species worldwide are affected by diverse diseases including trichomoniasis, a disease caused by the flagellated protist *Trichomonas gallinae* [1, 2]. Birds of prey that consume members of the family Columbidae are especially susceptible to trichomoniasis, as pigeons and doves are primary hosts for *T. gallinae* [3, 4]. Consequently, infectious diseases such as trichomoniasis rank among the most common causes of morbidity in some birds of prey [5]. Susceptibility to trichomoniasis varies among age classes in several species of predatory birds. In general, young birds are more likely to contract the disease than older birds, often leading to high rates of nestling mortality [2, 6, 7]. For example, trichomoniasis was identified as the cause of mortality in ca. 40% of nestlings produced annually in a population of urban-nesting Cooper’s hawks in Tucson, Arizona [2]. For susceptible Cooper’s hawks, infection by *T. gallinae* results in lesions in the mouth and oropharynx, with subsequent spread of lesions to the esophagus and crop [8]. Reduced food consumption and intermittent regurgitation resulting from severe lesions leads to weight loss and ultimately can be fatal [9].

Previous research suggests that susceptibility of Cooper’s hawks to trichomoniasis is related strongly to the age-specific pH of the oral cavity [10]. The mean pH of fluid in the oral cavity of nestling Cooper’s hawks is 6.8, whereas that of fledglings and adults is 6.0–6.1 (i.e., is at least seven times more acidic) [10]. Trichomonads generally are sensitive to environmental pH (e.g., *T. gallinae* [11], *T. gallinae* and *T. vaginalis* [12], *T. vaginalis* [13], *T. foetus* [14]). *Trichomonas gallinae* thrives when pH is between 6.5 and 7.5 (optimum 7.2 [11, 12]). When the pH is near optimum for trichomonads, some species (e.g., *T. vaginalis*) secrete a substance that facilitates invasion of host epithelial cells [15], but the ability to do so declines rapidly when pH is above or below the optimum.

Fluid in the oral cavity of Cooper’s hawks becomes more acidic after birds have fledged and are nearing independence (≥50 days of age; [10]), but the reason for the change is unknown. Many animals undergo similar changes in body chemistry (i.e., a change in acidity) during maturation, and those changes often are associated with changes in their bacterial communities [16–22]. We speculated that the community of bacteria in the oral cavity of nestling Cooper’s hawks differs from that in older hawks. Our objectives in this study were to provide a first perspective on the bacterial communities associated with the oral cavity of a wild raptor, and to compare the oral microbiomes of apparently healthy Cooper’s hawks in three age classes, sampled contemporaneously from a shared geographic area.

## Methods

We conducted the field portion of the study in Tucson, Arizona from April to July 2015. We sampled the oral microbiome of hawks from three age groups: nestlings (14–28 days old), fledglings (55–120 days old), and breeding adults (> 1 year old). We used a bal-chatri trap [23] to capture fledglings and adult males, and a dho-gaza trap [23] to capture adult females. Nestlings were taken by hand from their nests. We differentiated males from females by diameter of the tarsometatarsus [24]. All hawks were captured from nests monitored in a long-term study of population dynamics [25]. The University of Arizona’s IACUC reviewed and approved all of the activities in our study that involved the capture and handling of adult, fledgling, and nestling Cooper’s hawks (Protocol 12–329). Sampling locations are shown in Table 1 and Additional file 1: Figure S1.

**Table 1:** Collection information

Hawk	USFWS ID	Sex	Age class	Capture date	Latitude (°N)	Longitude (°W)	Nest ID	Capture method	Metatarsus (mm)	Bacterial OTU
1	1084–07931	Male	Adult	24-Apr-15	32.240887	− 111.006524	Murrieta	Bal-chatri	4.8	72
2*	1084–07932	Male	Adult	24-Apr-15	32.20184	− 110.93199	Eastmore Park	Bal-chatri	4.65	55
3	1084–03031	Male	Adult	08-May-15	32.247686	−110.865418	Seneca	Bal-chatri	N/A	82
4	1084–07933	Male	Adult	08-May-15	32.265451	−110.835344	Camino Suerte II	Bal-chatri	4.2	101
5	1084–07934	Male	Adult	08-May-15	32.247896	−110.908971	Tucson Bot. Gardens	Bal-chatri	4.6	90
6	1115–04490	Female	Nestling	22-May-15	32.197589	−110.886582	Freedom Park II	Hand	5.6	79
7	1115–04491	Female	Nestling	22-May-15	32.197589	−110.886582	Freedom Park II	Hand	5.5	80
8	1084–07935	Male	Nestling	22-May-15	32.197589	−110.886582	Freedom Park II	Hand	4.73	86
9	1115–04492	Female	Nestling	22-May-15	32.197589	−110.886582	Freedom Park II	Hand	5.98	66
10	1115–04493	Female	Nestling	26-May-15	32.240887	−111.006524	Murrieta	Hand	5.83	91
11	1115–04494	Female	Nestling	26-May-15	32.240887	−111.006524	Murrieta	Hand	5.27	68
12	1084–07936	Male	Nestling	26-May-15	32.240887	−111.006524	Murrieta	Hand	4.86	79
13	1084–07937	Male	Nestling	26-May-15	32.240887	−111.006524	Murrieta	Hand	4.04	99
14	1084–07938	Male	Nestling	26-May-15	32.240887	−111.006524	Murrieta	Hand	4.61	71
15	1115–04495	Female	Nestling	26-May-15	32.230845	−110.956559	Campus	Hand	5.62	88
16	1115–04496	Female	Nestling	26-May-15	32.230845	−110.956559	Campus	Hand	5.83	83
17	1084–07939	Male	Nestling	29-May-15	32.262021	−110.981927	Evergreen I	Hand	4.38	107
18	1084–07940	Male	Nestling	29-May-15	32.262021	−110.981927	Evergreen I	Hand	4.63	98
19	1084–07941	Male	Nestling	29-May-15	32.262021	−110.981927	Evergreen I	Hand	4.42	60
20	NA	NA	Nestling	29-May-15	32.263028	−110.97943	Evergreen II	Hand	NA	92
21	NA	NA	Nestling	29-May-15	32.263028	−110.97943	Evergreen II	Hand	NA	94
22	NA	NA	Nestling	29-May-15	32.263028	−110.97943	Evergreen II	Hand	NA	86
23	1115–04497	Female	Adult	10-Jun-15	32.234104	−110.931758	Himmel	Dho-gaza	5.7	108
24	1115–04498	Female	Adult	10-Jun-15	32.20184	−110.93199	Eastmore Park	Dho-gaza	5.5	108
25	1115–04499	Female	Adult	16-Jun-15	32.264643	−110.912126	Chapel	Dho-gaza	5.7	101
26	1115–04500	Female	Adult	16-Jun-15	32.248348	−110.892015	Hampton	Dho-gaza	5.8	91
27	1156–08183	Female	Adult	19-Jun-15	32.263028	−110.97943	Evergreen II	Dho-gaza	6.6	80
28	1156–08184	Female	Adult	19-Jun-15	32.265875	−110.980614	Evergreen III	Dho-gaza	6.5	90
29	1084–07942	Male	Fledgling	02-Jul-15	32.264643	−110.912126	Chapel	Bal-chatri	4.3	72
30	1084–07943	Male	Fledgling	02-Jul-15	32.239478	−110.889069	Swanway Park II	Bal-chatri	4.04	65
31	1156–08185	Female	Fledgling	07-Jul-15	32.20004	−110.953109	Mirasol	Bal-chatri	5.9	79

Details for free-living Cooper’s hawks for which the oral microbiome was sampled in Tucson, Arizona, USA. Thirty-four individuals were sampled, but high-quality DNA extractions were obtained for 31 individuals (listed here). Information for each individual includes the US Fish and Wildlife Service identification number (USFWS ID), sex, age class, capture date, capture location, capture method, diameter of the metatarsus, and the number of operational taxonomic units (OTU) of bacteria recorded from swabs of the palate and tongue. NA, not applicable: individuals were too young to have been assigned a USFWS ID, identified to sex, or measured for metatarsus diameter. Asterisk indicates one individual excluded from richness and community analyses due to low bacterial richness

### Sampling the oral microbiome

We used a sterile foam-tipped applicator (Whatman, model WB100032) to swab the tongue and palate of each hawk. Prior to swabbing, we used scissors to trim the foam tip so it fit easily in the oral cavity. Scissors were soaked thoroughly with 95% ethanol prior to each use. We wore latex gloves and used a wooden tongue depressor to open the mouths of adult hawks. We used a new depressor for each hawk. Immediately after swabbing, we stored each foam tip in a sterile, 2 mL Eppendorf tube. We stored all tubes at − 80 °C within 2 h of sampling. In total we sampled 34 hawks; of these, 31 yielded high-quality DNA extractions and were included in our analyses (Table 1).

### Field controls

We used two types of controls in the field that allowed us to distinguish between bacterial associates of the oral cavity of hawks and those that might be captured incidentally during our sampling process. First, at each sampling event we retained a foam applicator that had been trimmed with scissors and exposed to the air as above, but not exposed to a hawk (field, air control). Second, we used an applicator that was trimmed as described above to swab a section of each tongue depressor that had not contacted a hawk or a person (field, wood control). These field-control swabs were processed contemporaneously with oral swabs as described below.

### DNA extractions

We used half of each foam tip for DNA extraction, retaining the second half for archival purposes. We used the PowerSoil DNA Isolation Kit (Mo Bio Laboratories: Carlsbad, California, USA) to extract total genomic DNA from each half-tip. We followed the manufacturer’s instructions, except that we added an initial incubation for 10 min at 65 °C in lysis buffer. We used the Qubit 2.0 Fluorometer (Invitrogen: Carlsbad, California, USA) with the Qubit dsDNA HS (High Sensitivity) Assay Kit to quantify DNA concentrations following the manufacturer’s instructions.

### Library preparation and Illumina sequencing

We used a dual-barcoded, two-step PCR approach for high throughput amplicon sequencing on the Illumina MiSeq platform (Illumina, Inc.) [26]. In the first PCR (PCR1) we amplified the V4 region of the 16S rRNA with primer pair 515F/806R [27]. Each PCR1 primer contained a universal 22 basepair (bp) consensus sequence tag (i.e., CS1 forward and CS2 reverse), 0–5 bp for phase-shifting, a 2 bp linker, and the locus-specific primer (515F or 806R). We pooled forward or reverse PCR1 primers with different phase-shifting lengths in equimolar concentrations prior to PCR, such that each amplification consisted of a random mixture of different phase-shifting lengths. This allowed for the maximum amount of sequence diversity in the first four bases, which is critical for accurate cluster identification and color matrix estimation on the Illumina MiSeq [28]. We chose linkers and phase-shifting bases with low identity between primers and the target sequences.

In the second amplification step (PCR2) we used forward and reverse primers that each contained the complement of the CS tag, a 12 bp barcode, and the corresponding Illumina sequencing primer. The addition of barcodes in PCR2 allowed for maximum flexibility in the locus of interest or to include multiple targets in the same sequencing reaction, without needing to purchase a large number of barcoded target-specific primers.

We performed PCR1 in triplicate in 15 μl reaction volumes that each contained 0.5 μl DNA template, 7.5 μl 1X Phusion Flash High-Fidelity PCR Master Mix (ThermoFisher Scientific, Austin, Texas, USA), 0.2 μl of 50 μM forward and reverse primers (505F and 806R respectively) [27], 1 mg/mL of molecular grade bovine serum albumin (BSA; New England Biolabs, Ipswich, Massachusetts, USA), and 6.0 μl of molecular grade water. We used the following cycling protocol: an initial denaturing step at 98 °C for 10 s; 25 cycles consisting of denaturation at 98 °C for 1 s, annealing at 57 °C for 5 s, and extension at 72 °C for 20 s; and a final extension at 72 °C for 1 min. We used sterile, molecular grade water instead of template for negative controls.

We used SYBR Green 1 (Molecular Probes, Invitrogen) to visualize amplification on a 2% agarose gel after electrophoresis. We then pooled the three PCR1 products for each sample. From this, we diluted 5 μl of the pooled amplicons with molecular grade water to a final concentration of 1:15. We then used 1 μl of the pooled, diluted PCR1 product as the template for PCR2.

Each PCR2 reaction contained a final concentration of 1X Phusion Flash High-Fidelity PCR Master Mix, 0.075 μM barcoded primers (forward and reverse previously pooled at a concentration of 2 μM), and 0.24 mg/mL of BSA, for a final volume of 20 μL. We used the following cycling protocol: an initial denaturing step at 98 °C for 10 s; five cycles consisting of denaturation at 98 °C for 1 s, annealing at 51 °C for 5 s, and extension at 72 °C for 15 s; and a final extension step at 72 °C for 1 min.

We visualized products of PCR2 on a 2% agarose gel to verify amplification with minimal primer dimers. We quantified PCR2 products with PicoGreen and the Biotek Synergy H1 Multi-Mode Reader (Winooski, Vermont, USA). We normalized amplicons to 1 ng/μL and pooled 2 μL of each for Illumina sequencing. We purified the final amplicon pool with Agencourt AMPure XP beads (Beckman Coulter, Inc., Brea, California, USA) at a ratio of 1:1 to remove excess primers, nucleotides, salts, enzymes, and primer dimers, following the manufacturer’s instructions.

We evaluated the amplicon library on a BioAnalyzer 2100 (Agilent Technologies) to determine concentration and fragment size distribution. Paired-end sequencing was performed on an Illumina MiSeq with Reagent Kit v3 (2 × 300 bp) according to protocols at the IBEST Genomics Core at the University of Idaho.

### Laboratory controls

We complemented our field controls (i.e., air and wood controls) with several precautionary measures in the laboratory to limit contamination. We prepared PCR mixes in a sterile, dedicated “pre-PCR” hood, which never was exposed to amplified DNA. We decontaminated pipettes and all surfaces in the pre-PCR hood with DNA Away (Molecular Bioproducts, Inc., San Diego, California, USA) prior to each use. We used a dedicated “post-PCR” hood for all pre-sequencing steps after PCR1 (i.e., PCR1 pooling, dilutions, addition of diluted products to PCR2 master mix, and PCR2 amplicon pooling). We used DNA Away and treated the hood and all equipment with ultra-violet light for a minimum of 30 min prior to each use. We used sterile, aerosol-resistant pipette tips at all steps to minimize cross-contamination of samples, and we used separate reagents, pipettes, tips, and consumables for pre- and post-PCR setup. We pooled negative controls from PCR1 and used them as template for reactions in PCR2 to ensure that no contamination occurred during pooling or PCR2 setup. Finally, although we detected no contamination, we combined 5 μL of each PCR negative control, extraction blank, and field control in a separate pool and subjected them to the same pre-sequencing treatment as positive amplicon pools. We sequenced these negative controls in parallel with our samples.

### Mock communities

To measure error rate, determine the consistency of quality among replicates, assess the correlation of operon number and read count, and filter sequence data, we amplified and sequenced bacterial mock communities in parallel with our samples. The mock communities were obtained through BEI resources (American Type Culture Collection, Manassas, Virginia, USA), the National Institute of Allergy and Infectious Diseases, and the National Institutes of Health as part of the Human Microbiome Project. We used two preparations: Genomic DNA from Microbial Mock Community B (Even, Low Concentration), v5.1 L, for 16S rRNA Gene Sequencing, HM-782D; and Genomic DNA from Microbial Mock Community B (Staggered, Low Concentration), v5.2 L, for 16S rRNA Gene Sequencing, HM-783D. Each preparation contained 20 phylogenetically diverse bacterial taxa either at even concentrations of 100,000 copies per organism per μL (even mock community) or staggered concentrations from 1000 to 1000,000 copies per organism per μL (staggered mock community). We amplified 1 μL of each mock community for PCR1 as above. To assess within-run variation, we used 1 μL of diluted PCR1 product (even and staggered) for five separate PCR2 amplifications, resulting in amplicons with five different barcodes for each mock community. Overall, we sequenced 10 replicate mock samples in parallel with oral swabs, field controls, and laboratory controls.

### Data preparation

We demultiplexed raw Illumina reads with custom scripts at the IBEST Genomics Core (https://github.com/msettles/dbcAmplicons) (one mismatch allowed in barcode, four mismatches allowed in primers with ends matching). Overall, we obtained 1,011,997 paired-end reads after demultiplexing. We used FastQC to assess the quality of demultiplexed reads (http://www.bioinformatics.babraham.ac.uk/projects/fastqc/).

We used the UPARSE pipeline with USEARCH v.8.1.1861_i86linux32 [29] for all subsequent analyses. We used the fastq_eestats2 command to create a summary report showing the number of reads that would pass an expected error filter (maxEE 0.25, 0.50, 1.0) at different length thresholds (length_cutoffs 200, 250, 10). Based on this report we chose a length cutoff that would yield a sufficient number of high quality reads per sample while also retaining maximum read length. We used the fastq_filter command to trim forward (R1) reads to 200 bp (fastq_trunclen = 200) and to remove reads with greater than one error rate (max_ee = 1). Overall, 952,214 reads passed this quality filtering (94%).

We used the command derep_fulllength to dereplicate quality-trimmed reads and then removed singletons (parameters -sizeout -minuniquesize 1). We clustered dereplicated sequences (48,221 sequences) into operational taxonomic units (OTU) at 97% sequence similarity. In addition to de novo chimera checking performed during clustering, we used the RDP classifier for reference-based chimera checking of representative sequences for each OTU (16S rRNA reference database v.9). We used the command–usearch_global (id = 0.97) to map raw reads back to 478 chimera-checked OTU. From the resulting OTU table we removed 48 OTU that represented potential contaminants (i.e., OTU present in the PCR negative controls (16), extraction blanks (4), or field controls (11), or spurious OTU found in the mock community (17)). We retained 430 OTU for analysis (Additional file 2: Table S1). Following quality control we retained high-quality data from 31 hawks (Table 1).

### Data analysis

We used the RDP naïve Bayesian 16S rRNA classifier to estimate taxonomy of each OTU. We used analyses of variance (ANOVA) to compare read number among age classes (nestlings, fledglings, and adults), sampling dates (Table 1), and sampling locations (Table 1). We used a t-test to compare read number between male and female hawks. We used a Kruskal-Wallis test to compare richness among age classes. We used ANOVA to compare read number for replicate analyses of the even mock community. We used linear regression to compare read number and operon number for the staggered mock community. Normality was confirmed before all parametric tests. We used analyses of similarity (ANOSIM) based on presence-absence data (Jaccard’s Index) and abundance data (Simpson’s Index) to evaluate community composition, implementing two sets of analyses: one that included only the OTU represented in the data set by ≥1000 reads (55 OTU), and one that included all nonsingleton OTU (236 OTU). We used a Bonferroni correction to correct for multiple comparisons in each analysis and non-metric multidimensional scaling implemented in PAST [30] to visualize results.

## Results

We obtained an average of 20,345 sequences from each hawk (95% CI: 15,252–25,437 sequences). Read number did not differ among nestlings, fledglings and adults (*n* = 31 hawks; log-transformed, F_2,28_ = 0.5154, *P* = 0.6028). Read number was consistent between males and females (t_29_ = − 0.2844, *P* = 0.7782), among sample-collection dates (*n* = 10 dates, log-transformed, F_9,21_ = 1.3857, *P* = 0.2559), and among sampling locations (*n* = 15 sampling locations, log-transformed, F_14,16_ = 0.8039, *P* = 0.6560). This consistency in read number permitted direct comparisons of richness among sample classes.

**Fig. 1:**
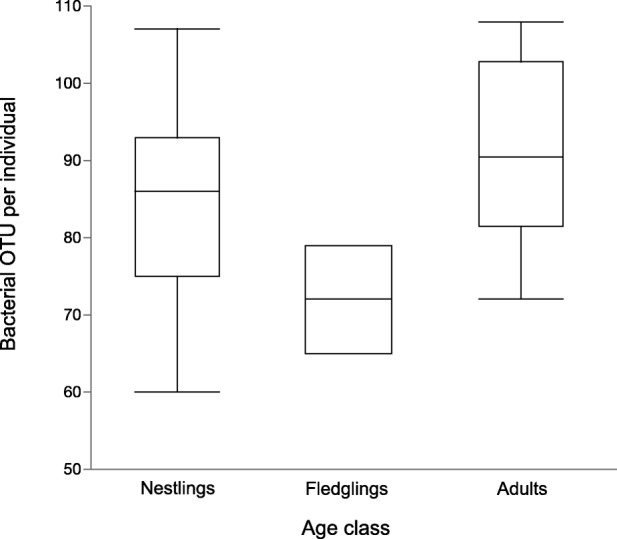
Bacterial richness in the oral cavity of individual Cooper’s hawks differed as a function of age class. Mean richness was lower in fledglings than in nestlings or adults. Results reflect data from 430 operational taxonomic units (OTU) that passed stringent quality control. Bars represent 95% confidence intervals for nestlings and adults; variation was relatively low among fledglings

We observed a mean of 84.5 bacterial OTU per individual hawk (95% CI: 79.3–89.7 OTU). One adult hawk was excluded from further analyses because richness from that sample was ≥2 standard deviations lower than the mean. Analysis of data from the remaining 30 hawks showed that bacterial richness differed as a function of age class (Fig. 1) (χ^2^ = 6.51, df = 2, *P* = 0.0386). Mean richness ranged from 92.3 OTU for adults (95% CI: 83.6–101.0) to lower values in nestlings and fledglings (respectively: 83.4 OTU, 95% CI: 77.4–90.5; 72.0 OTU, 95% CI: 54.6–89.4) (Fig. 1).

We analyzed residuals from the previous analysis to determine whether richness differed as a function of sex, sampling date, or sampling location once differences due to age class were taken into account. We observed no difference in richness between male and female hawks (t_28_ = − 0.3657, *P* = 0.7173), among sampling dates (F_9,20_ = 1.3524, *P* = 0.2730), or among sampling locations (F_14,15_ = 0.5887, *P* = 0.8356).

Analyses of mock communities demonstrated the consistency of results among replicates when operon counts were equal (even mock community, Additional file 3: Table S2), and a strong correlation of read number with operon number when inputs were unequal (staggered mock community, R^2^ = 0.88; Additional file 1: Figure S2). The latter observation led us to use read count as a proxy for abundance in subsequent analyses, permitting analyses based on both presence/absence data and abundance.

Overall, 55 OTU that passed the quality-control process were represented by > 1000 reads. None was present in any controls from the field or laboratory. These OTU were primarily members of Firmicutes (31.5% of OTU), Actinobacteria (22.2%), Proteobacteria (20.4%), Bacterioidetes (18.5%), and Tenericutes (3.7%), with one representative each of Fusobacteria and the SR1 clade (Table 2). Clostridia, Gammaproteobacteria, Actinobacteria, and Bacteroidia were numerically dominant, together accounting for 54% of OTU (Table 2). *Porphyromonas* was prevalent along with Micrococcineae, Corynebacterineae, *Suttonella, Mycoplasma*, and Actinomycineae (Table 2). Analyses of these OTU revealed that sampling was statistically complete for all age classes (Additional file 1: Figure S3), providing the basis for comparisons among age classes (below).

**Table 2:** Bacteria that differ in read abundance as a function of age class

OTU	Frequency in fledglings vs. nestlings	Frequency in adults vs. nestlings	Phylum	Genus
OTU 163	F > N (114.9)	A > N (52.4)	Bacteroidetes	*Porphyromonas*
OTU 45	F > N (14.7)	A > N (14.9)	Bacteroidetes	*Porphyromonas*
OTU 2394	F > N (7.9)	A > N (9.5)	Actinobacteria	Corynebacteriaceae sp.
OTU 114	F > N (5.5)	A > N (5.6)	Actinobacteria	Dermabacteraceae sp.
OTU 79	F > N (5.2)	A > N (3.9)	Bacteroidetes	*Proteiniphilum*
OTU 17	F > N (4.4)	(2.3)*	Firmicutes	*Veillonella*
OTU 47	F > N (3.9)	A > N (3.0)	Firmicutes	*Peptoniphilus*
OTU 37	F > N (3.8)	A > N (3.2)	Bacteroidetes	*Bacteroides*
OTU 21	F > N (3.2)	A > N (3.7)	Bacteroidetes	*Porphyromonas*
OTU 28	F > N (3.1)	A > N (2.9)	Proteobacteria	*Kingella*
OTU 82	F > N (3.1)	A > N (5.7)	Firmicutes	*Parvimonas*
OTU 1497	F > N (2.7)	A > N (2.8)	Proteobacteria	*Suttonella*
OTU 4714	F > N (2.6)	A > N (4.4)	Actinobacteria	Corynebacteriaceae sp*.*
OTU 136	(0.5)‡	A > N (2.6)	Proteobacteria	*Oleiphilus*
OTU 168	(1.7)‡	A > N (2.6)	Firmicutes	*Eubacterium*
OTU 170	(2.0)‡	A > N (3.0)	Actinobacteria	*Actinomyces*
OTU 157	(1.9)‡	A > N (4.5)	Actinobacteria	*Jonesia*
OTU 152	(0.6)‡	A > N (5.2)	Proteobacteria	*Lonepinella*
OTU 160	(0.6)‡	A > N (3.1)	Mollicutes	*Mycoplasma*
OTU 53	(0.4)‡	A > N (5.4)	Bacteroidetes	*Cruoricaptor*

Thirteen of the 55 most common operational taxonomic units (OTU) were ≥ 2.5 times more common in fledglings (F) than in nestlings (N) (marked F > N in the fledglings vs. nestlings column, with the fold-difference in read number shown in parentheses). Of these, 12 also were more common in adults (A) than in nestlings (N) (marked A > N in the adults vs. nestlings column, with the fold-difference in read number shown in parentheses). OTU 17 was 2.3-fold more common in adults vs. nestlings and is marked with an asterisk. Seven OTU were at least 2.5 fold more common in adults than in nestlings, but were not common in fledglings (OTU 136, 168, 170, 157, 152, 160, 53, marked with ‡), potentially reflecting the relatively small number of fledglings sampled here and consistent with the lower richness observed in fledglings (Fig. 1)

**Fig. 2:**
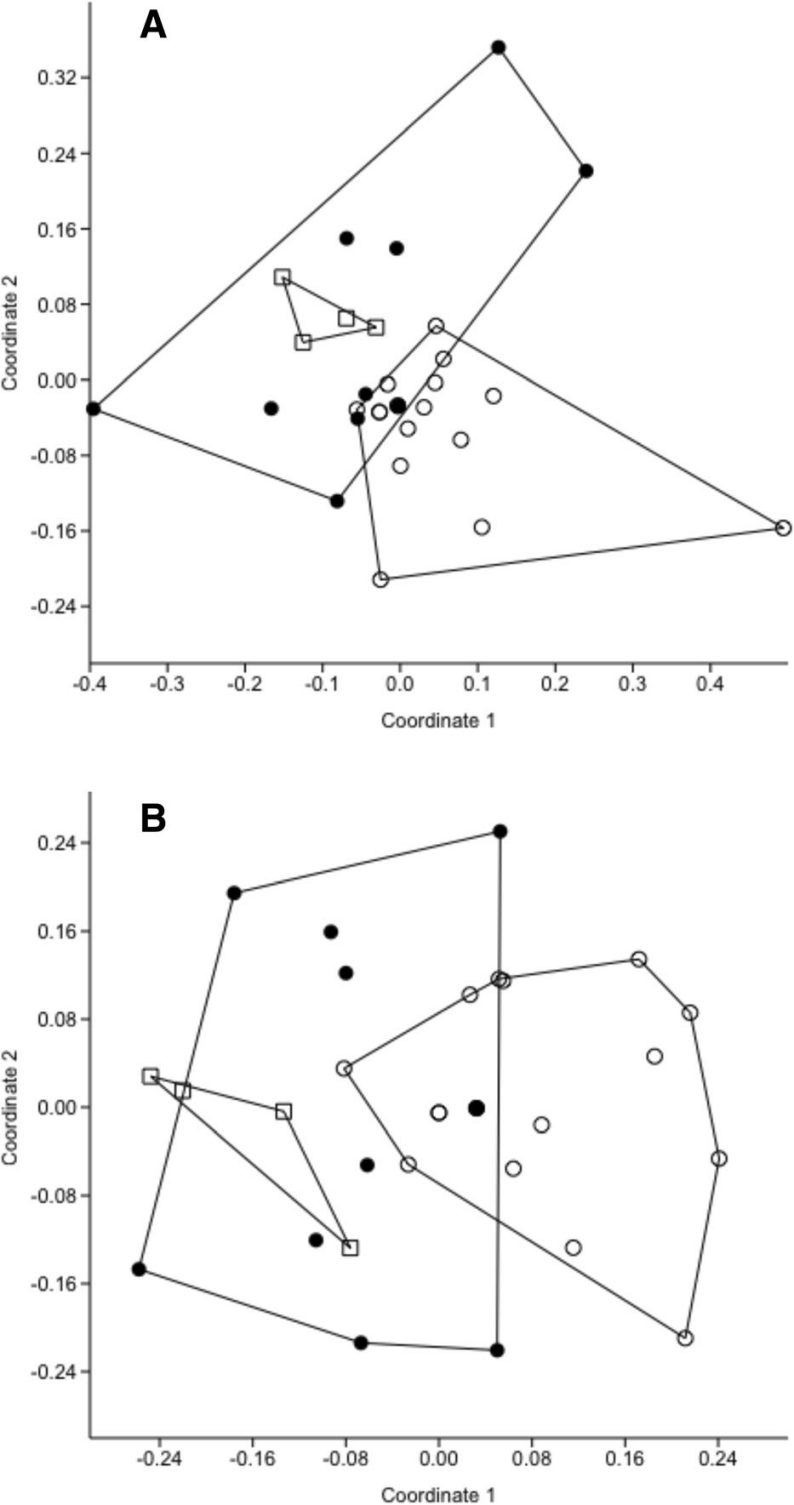
Communities of bacteria in the oral cavity differed as a function of age class in Cooper’s hawks. Non-metric multi-dimensional scaling analyses of the 55 most common operational taxonomic units (OTU) reveal that communities of bacteria in the oral cavity differ between nestlings (open circles) and more mature age classes (fledglings, open squares; adults, filled circles), which in turn did not differ from each other. Results are consistent when evaluated using **(a)** presence-absence data (Jaccard’s Index) or **(b)** read number as a proxy for abundance (Simpson’s Index)

When analyzed in terms of bacterial OTU, bacterial communities from the oral cavities of nestlings differed from those of fledglings and adults, whereas communities in fledglings and adults did not differ from each other (Fig. 2). Results were consistent when based on presence-absence data (Jaccard’s Index: ANOSIM R = 0.2059, *P* = 0.0168; Bonferroni-corrected pairwise comparisons: fledglings vs. nestlings, *P* = 0.0414; adults vs. nestlings, *P* = 0.0128; adults vs. fledglings, *P* = 0.9399) and abundance data (Simpson’s Index: ANOSIM R = 0.1925, *P* = 0.0015; Bonferroni-corrected pairwise comparisons: fledglings vs. nestlings, *P* = 0.0031; adults vs. nestlings, *P* = 0.0083; adults vs. fledglings, *P* = 0.6955). Analyses based on the full data set without singletons yielded the same results (Additional file 1: Figure S4).

To assess the quality of our inference, we evaluated whether differences in community structure among age classes could instead reflect differences in the sex of individuals (i.e., males vs. females) or the timing of sampling events. However, analyses restricted to fledglings and adults revealed that the oral microbiota of female and male hawks was similar (analysis of 55 OTU, as above; Jaccard’s index, ANOSIM R = 0.0583, *P* = 0.1743; Simpson’s index, R = 0.0306, *P* = 0.3171). Bacterial communities in these age classes did not differ as a function of sampling month (Table 1; Jaccard’s Index, ANOSIM R = 0.1181, *P* = 0.1363; Simpson’s Index, R = 0.0880, *P* = 0.1931). Analyses based on the full data set without singletons yielded the same results (Additional file 1: Figure S4).

Read counts for 13 of the 55 most common OTU (i.e., 24.1%) were at least 2.5 times as common in fledglings than in nestlings (Table 2). Twelve of these were also at least 2.5 times as common in adults than in nestlings (Table 2). These 12 OTU included putative members of the genera *Porphyromonas, Proteiniphilum, Veillonella, Peptoniphilus, Kingella, Parvimonas,* and *Suttonella,* as well as representatives of the Corynebacteriaceae and Dermabacteraceae (Table 2). Seven additional OTU were at least 2.5 times as common in adults vs. nestlings but were not as common in the transitional fledgling stage, although sampling of the fledgling age class was relatively limited (Table 2).

## Discussion

The oral microbiota of urban Cooper’s hawks differs between nestlings and older individuals. Differences in the oral microbiota could not be attributed to sampling month, location, or the sex of birds. These differences correspond to previously detected differences in the oral pH of Cooper’s hawks [10]. It is unclear whether the previously documented change in oral pH between nestlings and older birds could be attributed to an increase in the abundance of acid-producing bacteria as birds mature, or whether the shift is endogenous and selects for the establishment of acidophilic and/or acid-producing bacteria in older individuals. However, we observed that communities in the oral cavities of fledgling and adult hawks have high abundances of acid-producing bacteria, providing a basis for further study.

### Comparison of the oral microbiome of Cooper’s hawks relative to that of other vertebrates

The oral microbiome of Cooper’s hawks included bacterial phyla that are prevalent in the oral cavity of other vertebrates. The most common OTU in the present study represented Firmicutes, Actinobacteria, Proteobacteria, and Bacteroidetes. Firmicutes, Proteobacteria, and Bacteroidetes are especially common in the oral microbiome of healthy dogs [31]. These phyla also predominate in the oral microbiome of domestic cats [32, 33] and humans [34], wherein Spirochaetes, Actinobacteria, and Synergisetes also are common. As in those vertebrates, *Porphyromonas* was particularly prevalent in the oral microbiome of Cooper’s hawks, especially in older age classes (Table 2).

### Previous studies on bird-associated microbiota have not focused on the oral cavity

Previous work on the microbiomes of birds has focused primarily on cloacal [35], gut [36–40], and facial microbiomes [40]. Studies of vultures suggest an oral-gastrointestinal-fecal route for *Clostridia* and other microbial taxa, consistent with our observations of these bacteria in the oral cavity of Cooper’s hawks [40]. Many of the dominant genera observed here are more consistently found in oral cavities of other vertebrates than in studies of other bodily microbiomes in birds [31–40].

### Age-specific variation in the oral microbiota

Several studies focusing on microbial communities associated with birds have explored bacterial community composition as a function of age. For example, the gut microbiota of juvenile kakapos (*Strigops habroptilus*) differed from that of adults in the prevalence of *Lactobacillus*, but communities did not differ otherwise among individuals of different ages [41]. Cloacal microbiomes differed significantly between chicks and adults of kittiwakes (*Rissa tridactyla*) [35]. In poultry such as chickens (*Gallus gallus domesticus*), major changes in the cecal microbiome are observed several weeks after hatching relative to birds on the day of hatch [37]. Among 59 neotropical bird species, the age of individuals appears to be less important than taxonomic affiliation in predicting the composition of the gut microbiome, suggesting that age-related structure is more easily observed within bird species, rather than among them [36].

We found that nestlings differed markedly in the composition of their oral microbiomes relative to adults and fledglings. This difference is striking given the feeding behavior of this species. Early in the nestling phase, Cooper’s hawks are fed by the adult female. She tears pieces of flesh from prey items delivered by the adult male and individually feeds each nestling. Late in the nestling phase, chicks are strong enough to dismantle prey delivered to the nest on their own. After fledging, prey items are delivered whole to fledglings who usually are perched near the nest. Prey species delivered to nestlings in Tucson are dominated by birds, 57% of which are members of the Columbidae [42]. Breeding adults consume the same prey items they feed their nestlings, so it is unlikely that changes in diet among age classes play a role in the changes in oral microbiomes we observed.

### Taxonomic composition and potential relevance to the pH of the oral cavity in Cooper’s hawks

Taxa such as *Porphyromonas, Proteiniphilum, Parvimonas, Kingella, Suttonella, Peptoniphilus,* and *Veillonella* were especially common in samples from fledglings and adult hawks but were less commonly observed in nestlings. *Porphyromonas* species are known to produce acidic products (e.g., butyric acid) [43]. *Proteiniphilum* strains grow at a pH similar to that of the oral microbiome of fledgling and adult hawks (ca. 6.0) and can produce acetic acid [44]. *Parvimonas* strains metabolize peptone and amino acids to form acetic acid [45]. Acid production is known in *Kingella, Suttonella,* and *Peptoniphilus* species [46–48], and *Veillonella* strains can produce acetic and proprionic acids [49] (also characteristic of some Corynebacteriaceae, Table 2). Although it is unclear whether these bacteria are responsible for the age-related change in pH in the oral microbiome of Cooper’s hawks, the results are consistent with observed differences in pH reported previously [10]. In future work, isolating culturable members of the oral microbiota may be useful to support experiments evaluating whether it is pH per se, or microbial community composition, that correlates with the age-specific differences in susceptibility to *T. gallinae* observed previously in Cooper’s hawks.

### Methodological considerations

We designed our study to maximize quality control in the sampling, molecular analysis, and bioinformatics steps. Analyses of mock communities demonstrated the consistency of runs and a strong correlation of read number with operon number when inputs were unequal, as is anticipated for natural communities. Use of multiple field controls, replicated sampling within each age class, phylogenetically diverse mock communities prepared in two different ways, diverse measures to limit contamination in the laboratory, stringent quality-control thresholds, analyses that considered the most thoroughly sampled subset of the microbial community as well as the community as a whole, and analyses based on both presence/absence and read abundance allowed us to overcome some of the challenges that can restrict the inferential quality of microbiome data sets. Overall, our detection of robust differences in community structure among bacteria of the oral cavity in nestling hawks vs. fledglings and adults, and our testing of alternative explanations for patterns observed here (e.g., sex-related differences or differences as a function of sampling month) provide support for our conclusions.

## Conclusions

Our observations of the age-structured oral microbiome of Cooper’s hawks are consistent with established, age-related differences in oral pH between nestlings and older individuals. Previous work has shown that distinctive microbial communities are associated with diseases caused by trichomonads (e.g., in the human vaginal microbiome [50]). Although further study is needed to evaluate causal relationships among microbiome composition, oral pH, and trichomoniasis, our study provides a first perspective on the bacterial communities associated with the oral cavity of a wild raptor and sets the stage for further research to explore how susceptibility to parasites such as *T. gallinae* may be placed in a microbial community context.

## Additional files

Additional file 1: **Figure S1.** Sampling locations in Tucson, Arizona, USA. Points are numbered by representative hawks, corresponding to hawk ID numbers shown in Table 1. Scale, 0.6 cm = 1 km. **Figure S2.** Strong positive correlation between read count and operon number for staggered mock community, used to confirm that read abundance could be used as a proxy for OTU abundance. **Figure S3.** Species-accumulation curve for the 55 most common OTU indicates thorough sampling of bacterial communities, providing a basis for statistical analyses presented in the text. **Figure S4.** Communities of bacteria in the oral cavity differed as a function of age class in Cooper’s hawks. Non-metric multi-dimensional scaling analyses of all nonsingleton OTU that passed quality control for nestlings, fledglings, and adults. Panel A, Jaccard’s Index; panel B, Simpson’s Index. (DOCX 1850 kb)

Additional file 2: **Table S1.** Summary of operational taxonomic units for oral microbiome of urban Cooper’s hawks. Excel file containing operational taxonomic units, taxonomy, and occurrence of each in individual hawks. (XLSX 115 kb)

Additional file 3: **Table S2.** Mock community, number of operons, and average reads observed for the even and staggered mock communities. Table containing details for each mock community. (XLSX 56 kb)

## Abbreviations

16S rRNA: 16S ribosomal ribonucleic acid
ANOSIM: analysis of similarity
ANOVA: analysis of variance
CI: confidence interval
DNA: deoxyribonucleic acid
NA: not applicable
OTU: operational taxonomic unit
PCR: polymerase chain reaction
USA: United States of America
USFWS ID: United States Fish and Wildlife Service identification number
