# Supplementary material for: Age-related variation in the oral microbiome of urban Cooper’s hawks (Accipiter cooperii)
Source: BMC Microbiol. 2019 Feb 21;19:47. doi: 10.1186/s12866-019-1413-y (PMC6385412; doi:10.1186/s12866-019-1413-y)
Supplement: Supplementary file 1 — Figure S1. Sampling locations in Tucson, Arizona, USA. Points are numbered by representative hawks, corresponding to hawk ID numbers shown in Table 1. Scale, 0.6 cm = 1 km. Figure S2. Strong positive correlation between read count and operon number for staggered mock community, used to confirm that read abundance could be used as a proxy for OTU abundance. Figure S3. Species-accumulation curve for the 55 most common OTU indicates thorough sampling of bacterial communities, providing a basis for statistical analyses presented in the text. Figure S4. Communities of bacteria in the oral cavity differed as a function of age class in Cooper’s hawks. Non-metric multi-dimensional scaling analyses of all nonsingleton OTU that passed quality control for nestlings, fledglings, and adults. Panel A, Jaccard’s Index; panel B, Simpson’s Index. (DOCX 1850 kb) [file 12866_2019_1413_MOESM1_ESM.docx]

**Supplementary figures**

**Supplementary Fig. 1. Sampling locations in Tucson, Arizona, USA.** Points are numbered by representative hawks, corresponding to hawk identification numbers shown in Table 1. Scale, 0.6 cm = 1 km.

**Supplementary Fig. 2. Strong positive correlation between read count and operon number for staggered mock community.**

**Supplementary Fig. 3. Species-accumulation curve** for the 55 most common OTU indicates thorough sampling of bacterial communities, providing a basis for statistical analyses presented in the text.

**Supplementary Fig. 4. Communities of bacteria in the oral cavity differed as a function of age class in Cooper’s hawks.** Non-metric multi-dimensional scaling analyses of all nonsingleton OTU that passed quality control for nestlings (open circles), fledglings (open squares), and adults (filled circles). Results are consistent when evaluated using presence-absence data (panel A, Jaccard’s index) or read number as a proxy for abundance (panel B, Simpson’s index). As in analyses of the 55 most common OTU, these analyses of all nonsingleton OTU reveal that bacterial communities in nestlings differ from those in fledglings and adults, which in turn do not differ from one another (Jaccard’s index, ANOSIM R = 0.1988, P = 0.0208; Simpson’s index, R = 0.2327, P = 0.0071; n = 31 and 29 hawks, respectively, with two adults removed from the latter analysis due to highly divergent communities separated from others by their prevalence of rare taxa). Further analyses of all nonsingleton OTU also reveal that bacterial communities did not differ between males and females (data not shown; Jaccard’s index, R =0.0904, P = 0.0830; Simson’s index, R = - 0.0719, P = 0.9008) nor as a function of sampling month (adults and fledglings only; Jaccard’s index, R = 0.0521, P = 0.2944; Simpson’s index, R = -0.0532, P = 0.6994; n = 14).

**Supplementary figure 1**

**Supplementary figure 2**


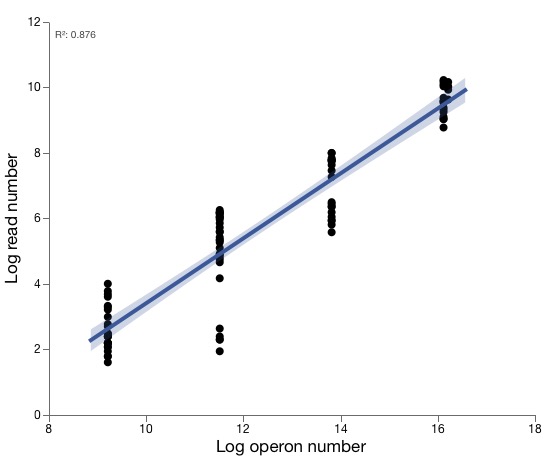


**Supplementary figure 3**

**
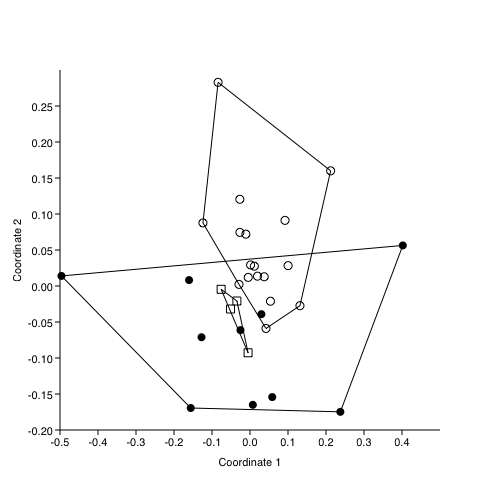
Supplementary figure 4**

**
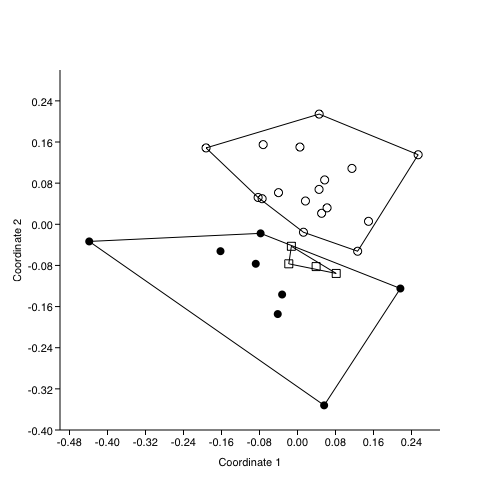
**

A

B
